# The impact of human milk oligosaccharides and galacto-oligosaccharides on the growth of early life pathogenic gut bacteria

**DOI:** 10.3389/fmicb.2026.1854651

**Published:** 2026-07-28

**Authors:** Job T. S. Schlösser, Wendy W. J. Unger, Ad C. J. M. de Bruijn, Dianne J. Delsing, Andre Groeneveld, Arjen Nauta, Gert Folkerts, Marc M. S. M. Wösten, Saskia Braber

**Affiliations:** 1 Division of Pharmacology, Utrecht Institute for Pharmaceutical Sciences, Faculty of Science, Utrecht University, Utrecht, Netherlands; 2 Laboratory of Pediatrics, Division of Pediatric Infectious Diseases and Immunology, Erasmus MC University Medical Center Rotterdam – Sophia Children's Hospital, Rotterdam, Netherlands; 3 Friesland Campina, Amersfoort, Netherlands; 4 Division of Infectious Diseases and Immunology, Department of Biomolecular Health Sciences, Faculty of Veterinary Medicine, Utrecht University, Utrecht, Netherlands

**Keywords:** antibiotic alternatives, bacterial growth, culture media, early life intestinal pathogens, galacto-oligosaccharides, human milk oligosaccharides

## Abstract

Antibiotics are widely used to prevent and treat early life gut infections, but their aberrant use has raised global concerns about antibiotic resistance. As breastfed infants have fewer intestinal infections than formula-fed infants, this stimulated scientific interest in the anti-pathogenic properties of breastmilk, specifically human milk oligosaccharides (HMOs). HMOs are non-digestible sugars that support infant gut microbiome development, immune maturation, and protection against pathogens. However, to determine how specific HMO structures differentially affect microbial growth and pathogen susceptibility investigating individual HMOs is essential.

This study therefore evaluates the effects of single-structure HMOs and galacto-oligosaccharides (GOS) on the growth of key early-life gut pathogens, *Escherichia coli, Salmonella enterica, Yersinia enterocolitica*, and *Clostridium perfringens*. The strongest pathogen inhibitory effects of HMOs were further assessed by colony forming unit enumeration and tested across variable media to confirm oligosaccharide-specific efficacy.

Oligosaccharide screening revealed clear differences in bacterial growth, with *S. enterica* most strongly inhibited and *C. perfringens* exhibiting growth stimulation. Among the tested oligosaccharides, 3′-galactosyllactose and GOS showed the most pronounced growth-inhibitory effects against *Escherichia coli, Salmonella enterica, Yersinia enterocolitica*. Salt concentration and nutrient richness further modulated oligosaccharide activity, highlighting the multifactorial nature of their antibacterial potential.

Overall, our findings show that oligosaccharides display structure-specific and environment-dependent effects on bacterial growth, indicating that their antimicrobial activity is shaped by oligosaccharide structure, bacterial species, and growth environment.

## 1 Introduction

At birth, the intestines are nearly sterile, but microbial colonization of the infant gastro-intestinal tract proceeds rapidly following delivery (Martino et al., 2022). The combination of an immature digestive and immune system with a developing gut microbiota renders neonates highly susceptible to gastrointestinal infections (Walsh et al., 2020; Singh et al., 2022). The infant intestinal microbiota remains less complex than that of adults until around age two, when it reaches an adult-like community structure (Martino et al., 2022). During this period, early life is particularly vulnerable to opportunistic pathogens, which can lead to diseases such as necrotizing enterocolitis (NEC) and gastroenteritis. Common clinical practice for managing these diseases involves both prophylactic and anaphylactic antibiotic treatments (Langdon et al., 2016; Hackam and Sodhi, 2022; Thänert et al., 2022). However, antibiotic exposure in early life can disrupt the gut microbiota, an ecosystem essential for normal digestion and immune maturation (Thänert et al., 2022). Such disturbances may hinder the establishment of healthy host-microbe interactions, with potential long-term consequences for overall health. Together with concerns regarding antibiotic overuse and the spread of antimicrobial resistance, these issues underscore the need to reduce antibiotic use and develop alternative preventive and therapeutic strategies (Darby et al., 2023; Murray et al., 2022; Ranjbar and Alam, 2023).

Human milk is the recommended exclusive nutrition during the first 6 months of life to support gastrointestinal maturation and providing essential nutrients (World Health Organization, 2019). However, lactation issues, maternal drug administration, insufficient milk composition and maternal personal choice often necessitate partial or complete replacement of human milk with infant formula. Comparative studies have reported a higher incidence of gastrointestinal infections in formula-fed than in breast-fed infants, underscoring the significance of bioactive constituents in human milk absent in formula milk (Borewicz et al., 2020; Inchingolo et al., 2024; Benno et al., 1984). Following lactose and lipids, human milk oligosaccharides (HMOs) represent the third most abundant solid constituent in human milk, occurring at concentrations of approximately 5 to 15 g/L. HMOs are non-digestible sugar chains composed of 3–10 saccharide units (Bode, 2020). These sugar chains consist of a lactose backbone decorated with various monosaccharides, including glucose, galactose, N-acetylglucosamine, fucose, and sialic acid. The abundance and composition of maternal HMO production vary according to genetic, environmental, and lactation-related factors, with the fucosylated HMOs generally predominating the composition (Bode, 2020; Soyyilmaz et al., 2021; McDonald et al., 2022). HMOs contribute to the development of the early-life gut ecosystem and are well known to promote the growth of beneficial bacteria, such as *Bifidobacterium* spp., whereas most pathogenic bacteria cannot efficiently utilize these compounds (Singh et al., 2022; Buzun et al., 2024; Yang et al., 2024). This selective nourishment supports a healthy microbial community and contributes to the infant's immune defense (Walsh et al., 2020; Singh et al., 2022). Furthermore, HMOs exhibit antibiofilm activity against several bacterial pathogens and attenuate bacteria-induced oxidative stress in the host (Bhowmik et al., 2022; Cai et al., 2022). The anti-microbial effects have primarily focused on pooled HMOs, demonstrating their antimicrobial activity against various pathogens, with some individual HMOs shown to specifically target *Streptococcus agalactiae* (Asadpoor et al., 2020; Ackerman et al., 2017; Craft et al., 2018; Lin et al., 2017). However, pathogens linked to early-life gut diseases, such as NEC and gastroenteritis, have not yet been evaluated for their susceptibility to the antimicrobial effects of individual HMOs.

Oligosaccharides are easy applicable as dietary additives and HMO manufacturing is currently an emerging field (Bührer et al., 2022; Chen, 2015). Galacto-oligosaccharides (GOS) are the most widely used oligosaccharide ingredient in infant formula, with proven beneficial effects on gut microbiota composition and gut health (Van Leeuwen et al., 2016; Barnett et al., 2023). GOS is a complex mixture comprising over 100 oligosaccharides with varying galactose linkages and degrees of polymerization (typically DP 2–7), including 3′-galactosyllactose (3′-GL), 4′-galactosyllactose (4′-GL), and 6′-galactosyllactose (6′-GL), which are also present in human milk within the HMO fraction (Logtenberg et al., 2020; Newburg et al., 2016; He et al., 2014).

Here, we aim to investigate the antimicrobial activity of individual HMOs, GOS and HMO mixtures against key early-life gut pathogens, *Escherichia coli* and *Clostridium perfringens*, both associated with NEC, as well as the gastroenteritis-associated species *Salmonella enterica* and *Yersinia enterocolitica*. These findings suggest that small variations in HMO structure can have a major impacts on antibacterial activity against the pathogenic gut bacteria examined in this study.

## 2 Results

### 2.1 Impact of HMOs and GOS on the growth of pathogenic bacteria *E. coli, S. enterica, Y. enterocolitica*, and *C. perfringens*

**Figure 1:**
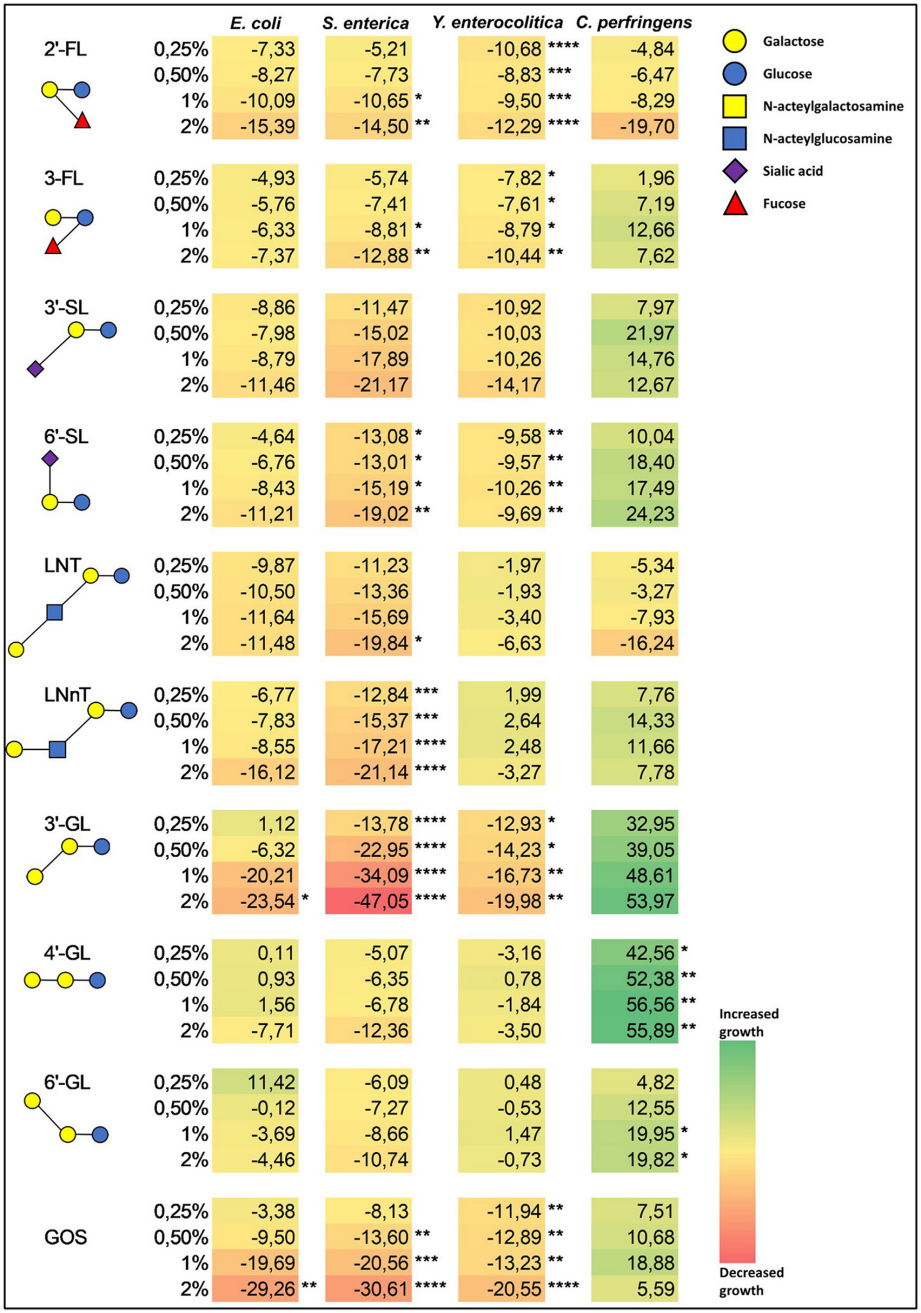
Heatmap depicting the percentage differences in the Area Under the Curve (AUC) between untreated and oligosaccharide-treated intestinal pathogens *E. coli, S. enterica, Y. enterocolitica*, and *C. perfringens*, based on 16-h growth curve. Red indicates a percentage decrease and green indicates a percentage increase in the AUC growth curve data of oligosaccharide-treated intestinal pathogens compared to untreated pathogens. Color intensities indicate the magnitude of the percentage difference. Each row represents different oligosaccharides at the concentrations: 0.25%, 0.5%, 1%, 2%. Each column represents the data from a single pathogen. All growth curves were performed in triplicates and repeated in at least three independent experiments. Statistical significance is indicated with *p-*value markers *, p<*0.05;* **, p<*0.01;* ***, p<*0.001;* ****, p<*0.0001*. For each HMO or GOS, the chemical structures are presented with the corresponding building block detailed in the legend at the top right. 2′-FL, 2′-fucosyllactose; 3-FL, 3-fucosyllactose; 3′-SL, 3′-sialyllactose; 6′-SL, 6′-sialylactose; LNT, lacto-N-tetraose; LNnT, lacto-N-neotetraose; 3′-GL, 3′-galactosyllactose; 4′-GL, 4′-galactosyllactose; 6′-GL, 6′-galactosyllactose.

To investigate whether HMOs and GOS possess antimicrobial activity, we monitored the growth dynamics of early-life gut pathogens *E. coli, S. enterica, Y. enterocolitica*, and *C. perfringens* in their respective growth media, both with and without 0.25%-2% HMOs or GOS, over a 16-h period. The area under the curve (AUC) was calculated for each growth curve and presented in a heatmap showing the percentage AUC difference between HMO/GOS-treated and untreated conditions (Figure 1).

A distinct growth difference was observed between the Gram-negative bacteria *E. coli, Y. enterocolitica*, and *S. enterica* and the Gram-positive bacterium *C. perfringens* in response to the oligosaccharides. The Gram-negative bacteria primarily demonstrated growth-reducing effects when incubated with individual HMOs and GOS. HMOs showed a trend toward increased growth of the Gram-positive *C. perfringens*, although this difference was only significant for 4′-GL and 6′-GL. Only a trend of growth reduction was demonstrated upon addition of lacto-N-tetraose (LNT) (−16.2%) or 2′-fucosyllactose (2′-FL) (−19.7%). Among the three Gram-negative bacteria, *S. enterica* was the most sensitive to nearly all HMOs tested. Especially, 2% GOS and 3′-GL reduced the growth of *S. enterica* by 30.6% and 47%, respectively. GOS and 3′-GL were the most active compounds against the Gram-negative pathogens. Interestingly, all three Gram-negative bacteria showed negligible growth inhibition to 4′-GL and 6′-GL which are structurally nearly identical to 3′-GL. In contrast, *C. perfringens* demonstrated growth-promoting effects in the presence of GLs, with a striking 56% increase in growth in the presence of 4′-GL. These results indicate that small structural variations in oligosaccharides can influence their growth-modulating effects across pathogenic species.

### 2.2 Detailed analysis of the growth-reducing impacts of 3′-GL and GOS on *E. coli, S. enterica* and *Y. enterocolitica*

**Figure 2:**
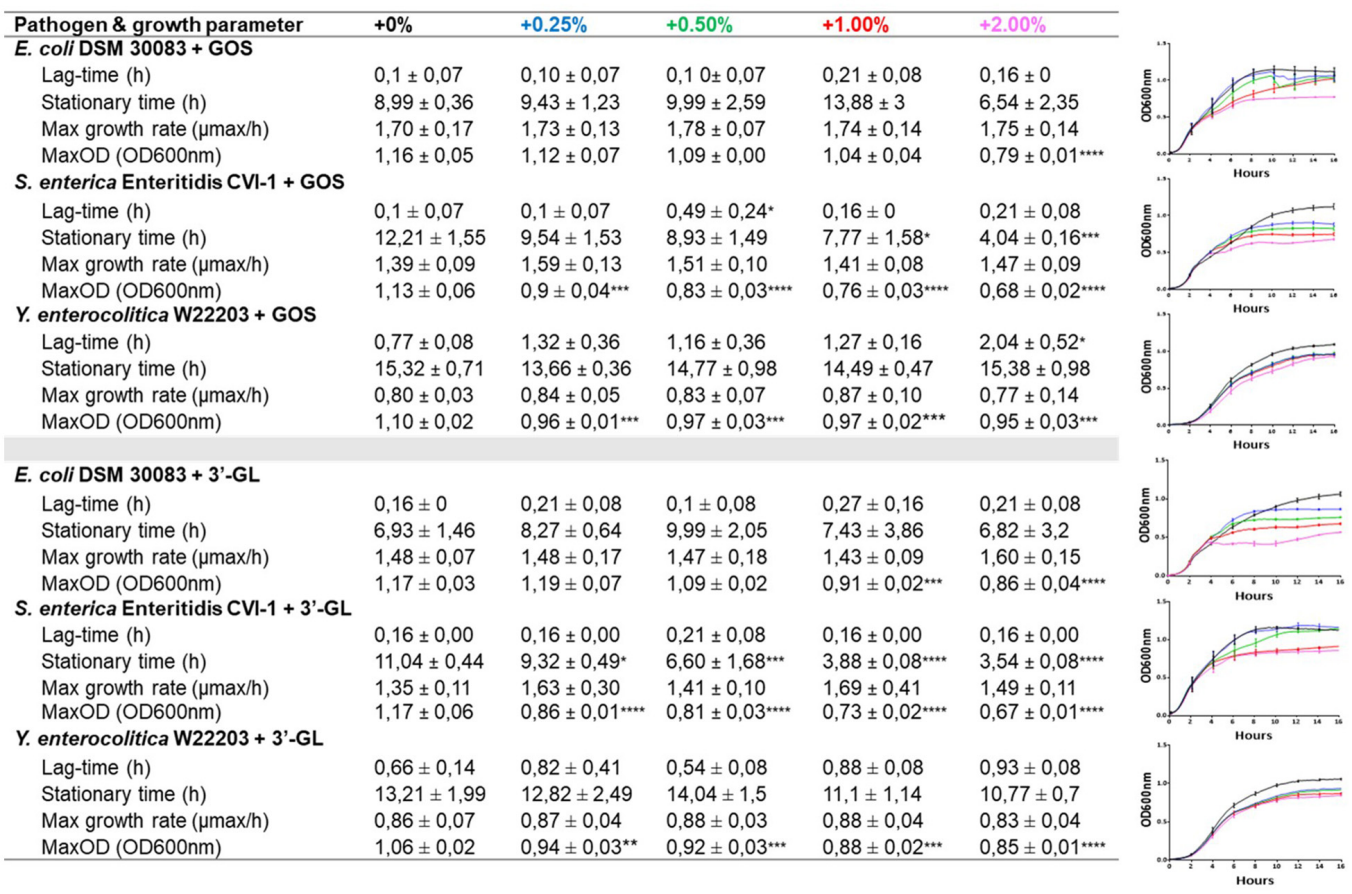
Growth curve parameter data and representative growth curves from *E. coli, S. enterica* and *Y. enterocolitica* + 0.25-2% GOS/3′-GL. To quantify these parameters, we adapted the algorithm described by Luisa Navarro-Pérez et al. (34). The growth parameter data of the generated 16-h optical density (600nm) growth curves with a 10 min interval are shown as mean ± SEM. The growth parameter data reflects, the end of the lag-time in hours, the start of the stationary time in hours, the maximum specific growth rate in maximum growth per hour and the maximum OD600nm reached. Next to the growth parameter data are the corresponding growth curves with SEM bars at every 2 h. The colors match with the displayed concentrations. All growth curves were performed in triplicates and repeated in at least three independent experiments. Data analysis was performed in triplicates and repeated in at least three independent experiments. Statistical significance is indicated by *p*-value markers *, p<*0.05;* **, p<*0.01;* ***, p<*0.001;* ****, p < 0.0001. h, hours; OD600nm, optical density 600nm; μmax, maximum specific growth rate.

To further assess the growth-reducing capacity of the highest impact oligosaccharides, 3′-GL and GOS, the growth curves were analyzed in detail by calculating key growth parameters: lag time (h), stationary phase time (h), maximum specific growth rate (μ*max/h*), and maxOD (OD600nm) (Figure 2).

As shown in Figure 2, the maxOD was consistently reduced by 3′-GL and GOS in the three analyzed bacteria, with *E. coli* showing only significant reductions at higher concentrations (2% GOS and 1–2% 3′-GL). The maximum growth rate was unaffected by 3′-GL and GOS in *E. coli, S. enterica* and *Y. enterocolitica*, while 2% GOS increased the lag-phase of *Y. enterocolitica*. Notably, *S. enterica* exhibited an earlier onset of the stationary phase in response to both 3′-GL and GOS, with GOS only effective at higher concentrations (1%-2%). Analysis of growth patterns indicates that 3′-GL and GOS inhibit bacterial growth primarily by limiting the maximum optical density reached, with an earlier onset of the stationary phase specifically observed in *S. enterica*.

### 2.3 The impact of GOS and 3′-GL on colony forming units of *E. coli, S. enterica* and *Y. enterocolitica*

To determine whether the reduced optical density observed in the growth curves corresponded to a lower bacterial count, GOS and 3′-GL (highest growth inhibiting potential) were further tested. Their potential impact on bacterial numbers was assessed through colony-forming unit (CFU) enumeration. Due to the limited availability and high cost of 3′-GL, CFU counts were performed only for *S. enterica* treated with 3′-GL (at 0, 4, 8 and 24h). *C. perfringens* was excluded from further analysis, as none of the tested HMOs significantly reduced its growth.

**Figure 3:**
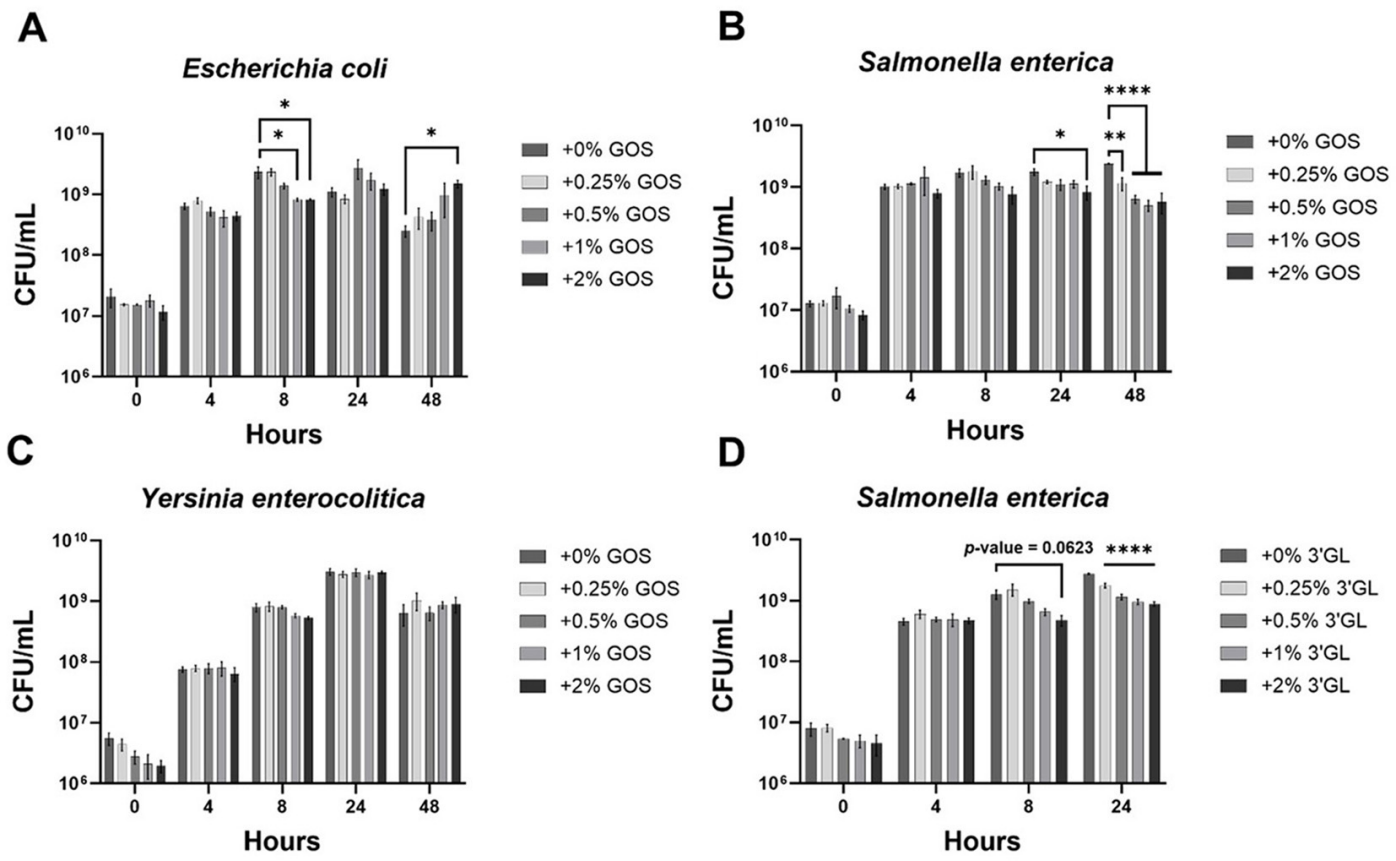
Colony forming unit (CFU) counts of *E. coli* + GOS **(A)**, *S. enterica* + GOS **(B)**, *Y. enterocolitica* + GOS **(C)**, and *S. enterica* +3′-GL **(D)** grown in Lennox LB medium and measured at 0, 4, 8, 24, (and 48h). Data are shown as mean ± SEM. All CFU experiments were performed in triplicates and repeated in at least three independent experiments. Statistical significance is indicated by *p*-value markers *, *p* < 0.05; **, *p* < 0.01; ***, *p* < 0.001; ****, *p* < 0.0001.

Increasing the GOS concentration in the culture medium to 1–2% resulted in a 3-fold reduction in *E. coli* CFUs after 8 h compared to the untreated control (8.2^*^ 10^8^ CFUs vs. 2.3^*^10^9^ CFUs). However, after 48 h, the opposite grow phenotype was observed, as 2% GOS supplementation resulted in a 6-fold increase of *E. coli* CFU counts (1.5^*^ 10^9^ CFUs vs. 2.5^*^10^8^ CFUs) (Figure 3A). For *S. enterica*, the presence of 2% GOS decreased CFU counts approximately 2-fold after 24 hours compared to the untreated control (8.3^*^10^8^ CFUs vs. 1.7^*^10^9^ CFUs). All tested 3'GL and GOS concentrations reduced *S. enterica* CFU counts after 24 hours and 48 hours, respectively (Figures 3B, D). In contrast, no significant effect of GOS on CFU counts was observed for *Y. enterocolitica* (Figure 3C). These results show that the effect of GOS supplementation on bacterial CFU count is dependent on sampling time. Moreover, a lower optical density caused by oligosaccharide-mediated inhibition, does not always correspond with a lower number of bacteria at the tested time-points.

### 2.4 Nutrient composition as well as salt concentrations modulates the antibacterial activity of GOS

**Figure 4:**
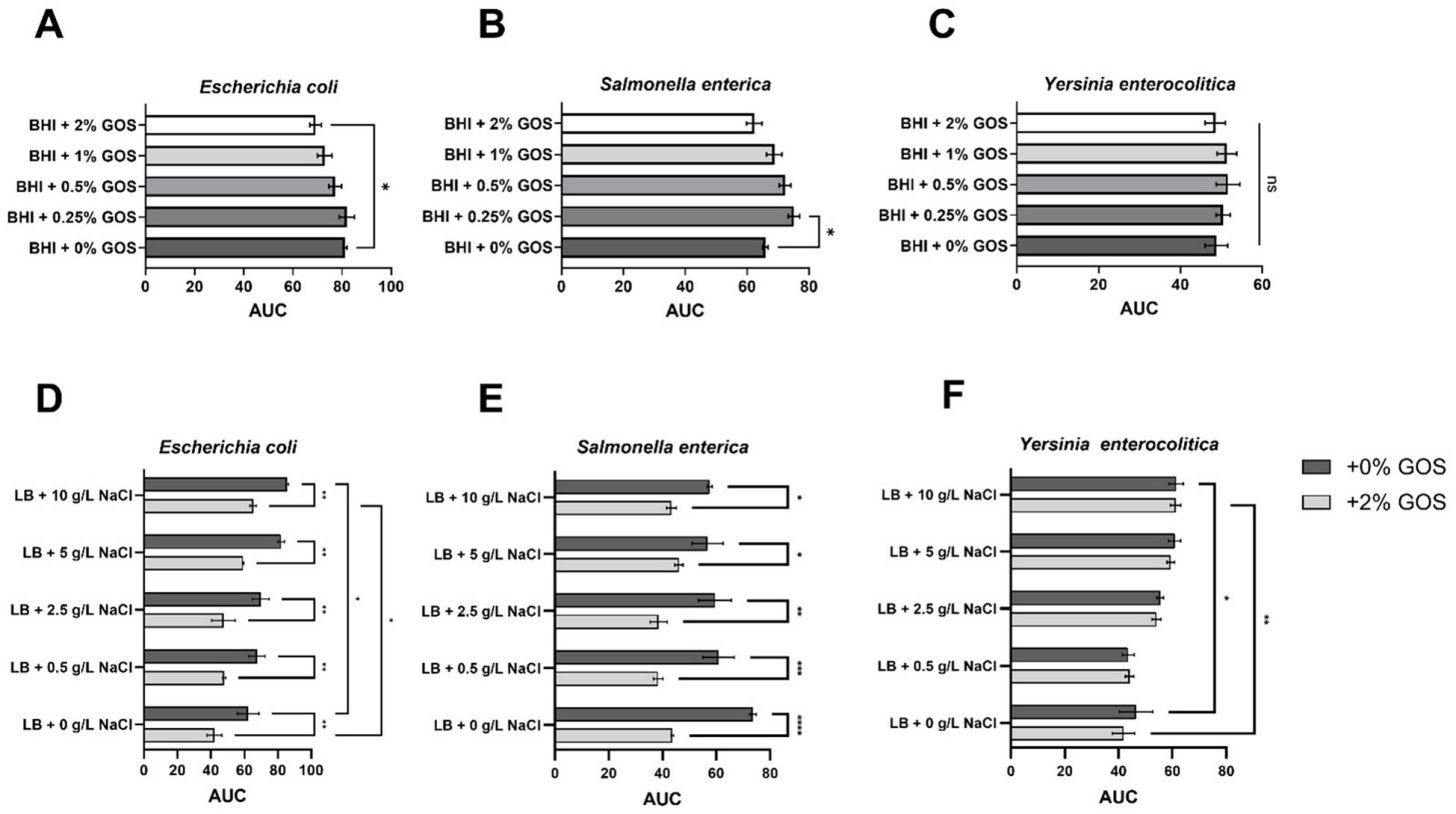
AUC data with SEM bars derived from 16-hour optical density growth curves in BHI medium from *E. coli* + GOS **(A)**, *S. enterica* + GOS **(B)** and *Y. enterocolitica* + GOS **(C)**. AUC data with SEM bars derived from 16-h optical density growth curves from *E. coli* **(D)**, *S. enterica* **(E)**, *Y. enterocolitica* **(F)** with and without 2% GOS in LB medium with a NaCl concentration ranging from 0 to 10 g/L. Growth curves were performed in triplicates **(A–C)** or duplicates **(D–F)** and repeated in at least three independent experiments. Statistical significance was assessed through one-way ANOVA with post hoc Tukey's test for multiple comparisons **(A–C)** or two-way ANOVA with post hoc Holm-Šídák's test for multiple comparisons **(D–F)** and indicated by p-value markers *, *p* < 0.05; **, *p* < 0.01; ***, *p* < 0.001; ****, *p* < 0.0001.

To investigate whether environmental factors, such as nutrient composition or salt concentration, influence the antimicrobial activity of GOS, we modified the growth medium or adjusted its salt concentration. To determine whether the observed antimicrobial effect is independent of the nutrient availability, the conventional culture medium Luria-Bertani (LB) medium was replaced with Brain Heart Infusion (BHI), which has a richer and more complex nutrient composition. The growth data are presented as AUC values calculated from 16-h optical density growth curves of *E. coli, S. enterica* and *Y. enterocolitica* treated with or without GOS (0.25–2%) in BHI medium (Figures 4A–C). *E. coli* grown in BHI medium demonstrated similar growth impacts in the presence of 2% GOS (Figure 4A). However, replacing LB with BHI medium attenuated the previously observed growth-inhibiting effects of GOS on *S. enterica* and *Y. enterocolitica* (Figures 4B-C). These data show that the available nutrients have a strong impact on the antibacterial activity of GOS.

Since human milk contains a relatively low salt concentration (0.2 - 0.5 g/L NaCl) (Bauer and Gerss, 2011) compared to the in this study used Lennox LB (5 g/L), we examined whether differences in salt concentration affect the impact of GOS on bacterial growth. To test this, *E. coli, S. enterica* and *Y. enterocolitica* were incubated with or without 2% GOS in LB medium containing NaCl concentrations from 0 to 10 g/L (Figures 4D–F).

The addition of NaCl alone to LB medium (with and without GOS) enhanced the growth of *E.coli* (Figure 4D) and *Y. enterocolitica* (Figure 4F), with a significant increase observed at 10 g/L NaCl compared to 0 g/L NaCl. On the other hand, *S. enterica* tended to grow less at higher NaCl levels in LB medium (Figure 4E). For *E. coli*, GOS inhibition remained stable across 0–10 g/L NaCl (Figure 4D), whereas *Y. enterocolitica* displayed no growth inhibition of GOS at any salinity levels. GOS inhibition of *S. enterica* was NaCl-dependent, showing greater effects at lower salt concentrations (Figure 4E). These data indicate that the antimicrobial activity of GOS not only depends on the nutrient composition but also on medium's salt concentration.

### 2.5 Influence of HMOs on biofilm formation and oxidative stress responses

As HMOs have been shown to reduce biofilms and oxidative stress responses, we evaluated the biofilm formation and oxidative stress responses of *E. coli, S. enterica* and *Y.enterocolitica* in the presence and absence of GOS. As depicted in Supplementary Figure 1A, no significant changes in biofilm formation were detected at any of the tested GOS concentrations across the three bacterial pathogens. Oxidative stress responses were assessed by monitoring hydrogen-peroxide mediated killing after 10 and 30 min of exposure to multiple H_2_O_2_ concentrations. The resulting CFU data (Supplementary Figure 1B) demonstrated no differences between the untreated and 2% GOS-treated conditions for any of the three bacterial pathogens, suggesting that GOS does not affect the oxidative stress response under the conditions tested.

### 2.6 HMO mixtures exhibit no growth-inhibitory effects against *E. coli, S. enterica* and *Y. enterocolitica*

**Figure 5:**
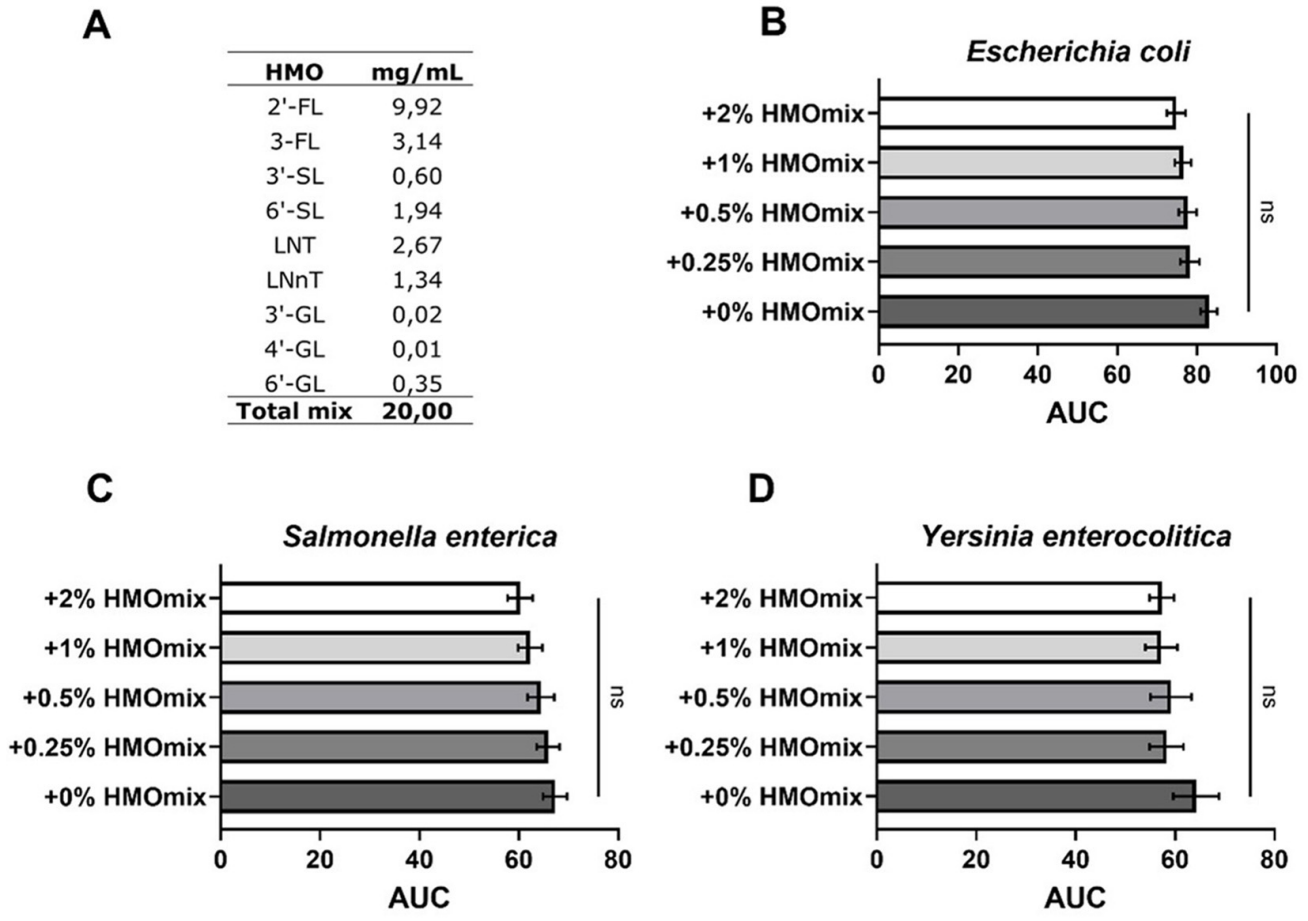
**(A)**, composition of HMO mixture prepared according to the described concentrations by Soyyilmaz et al. (24) and Newburg et al., (25). AUC data with SEM bars derived from 16-h optical density growth curves of pathogens *E. coli* **(B)**, *S. enterica* **(C)**, *Y. enterocolitica* **(D)** in LB medium with a NaCl concentration ranging from 0 to 10 g/L.

To evaluate potential additive or synergistic growth-inhibitory effects among the individual HMOs, a mixture of these HMOs was prepared. The HMO mixtures were prepared according to the average colostrum ratios reported by Soyyilmaz et al. (2021) and first-milk GL ratios established by Newburg et al. (2016) (Figure 5A).

Although increasing concentrations of the HMO mixture slightly reduce the growth of *E.coli, S. enterica* as well as *Y. enterocolitica*, the effects are not statistically different (Figures 5B–D). Overall, this specific HMO mixture exhibits only minor, if any, antimicrobial activity against these pathogens.

## 3 Discussion

This *in vitro* study evaluated the growth inhibitory potential of HMOs and GOS against early life relevant gut pathogens: *E. coli and C. perfringens*, associated with NEC and *S. enterica* and *Y. enterocolitica* associated with gastroenteritis.

The initial screening data displayed contrasting responses to individual HMOs and GOS between Gram-negative and Gram-positive bacteria: *E. coli, S. enterica* and *Y. enterocolitica* showed inhibited growth, whereas *C. perfringens* showed enhanced growth upon oligosaccharide treatment. Previous optical density data evaluating the individual HMOs and GOS growth impacts could not be directly compared to the Gram-negative vs. Gram-positive response patterns observed in this study (Lin et al., 2017; Craft et al., 2019; Mortaz et al., 2022; Renwick et al., 2025). GOS and 2′-FL showed similar capacity to reduce the growth of the Gram-positive *Staphylococcus aureus* as well as the Gram-negative bacterium *Pseudomonas aeruginosa* (Mortaz et al., 2022). Comparable growth-inhibitory effects of multiple individual HMOs were demonstrated against the Gram-positive bacterium *S. agalactiae* (Craft et al., 2018; Lin et al., 2017; Craft et al., 2019). The absence of consistent Gram-classification patterns in the growth-inhibitory response to oligosaccharide treatment may be due to the different bacterial species tested in the previous studies. This suggests that Gram classification alone is insufficient to predict bacterial sensitivity to oligosaccharides, and that species-level analysis is also required.

Based on the optical density data, almost all oligosaccharides revealed a growth-reducing capacity against *S. enterica*, especially 3′-GL was very effective. Similarly, *E. coli* and *Y. enterocolitica* exhibited specific sensitivity to 3′-GL, while showing only minor growth inhibition in response to the structurally related 4′-GL and 6′-GL. Another study investigating individual GL structures reported that 4′-GL, rather than 3′-GL, reduced the *E. coli* abundance within an established early life gut microbial community (Meeusen et al., 2024). The intricate microbial interplay, such as cross-feeding and nutrient competition within the established community, may underlie the observed discrepancy in responses to different GLs. In the study of Lin et al. (2017), structure-specificity was also observed for the inhibition of *S. agalactiae*. In this case, LNT strongly inhibited growth, whereas the structurally related compound lacto-N-neotetraose (LNnT) showed no inhibitory effect. These findings indicate that the growth inhibitory effects of HMOs may be explained by specific structures acting on specific bacterial species. This further emphasizes the importance of identifying HMO structure–microbe relationships, as defining specific structure-species interactions could support future research into direct anti-pathogenic effects and targeted therapeutic interventions.

Both 3′-GL and GOS significantly reduced growth of Gram-negative species, with growth pattern analysis identifying that a reduction in maximal optical density was the main contributing factor. Comparable GOS concentrations led to lower maximum optical densities in *S. aureus* and *P. aeruginosa*, consistent with our observations (Mortaz et al., 2022). The maximum optical density reflects bacterial efficiency in converting substrate into biomass. A lower value may indicate impaired or limited nutrient utilization (Bren et al., 2013). Potentially, the 3′-GL and GOS-induced growth inhibiting activity originate from less effective conversion from substrate to biomass. CFU measurements of the most growth reducing oligosaccharide–pathogen combinations did not consistently reflect the growth inhibition patterns observed in optical density data at every CFU time point (Figures 2 and 3). GOS reduced the growth rate of *E. coli* as estimated by CFU counting, but did not affect the maximum CFU count reached during the experiment (Figure 3A). In both the presence and absence of GOS, similar maximum CFU counts were reached, however, this maximum was reached earlier in the absence of GOS, indicating that GOS affects the growth rate rather than bacterial survival. In contrast, GOS reduced the CFU counts of *S. enterica* over a 48-h period, indicating an increasing effect on *S. enterica* survival over time. Similar inconsistencies between optical density data and CFU data have been reported in studies evaluating the effects of individual HMOs on *S. agalactiae*, including observations that HMOs affect the growth rate rather than the maximum CFU count over extended incubation periods (Craft et al., 2018, 2019). A plausible explanation is that oligosaccharides may affect biomass (monitoring bacterial cell expansion or monitoring dead cells) rather than actual bacterial cell numbers. Especially our *Y. enterocolitica* growth data, could indicate a decrease of biomass when treated with GOS without significant changes in bacterial cell counts (Figures 1 and 3C). On the other hand, *E. coli's* 3-fold reduction in cell number after 1–2% GOS treatment at 8-h did not align with the reduction of OD600nm (Figures 2 and 3A). Before attributing true antimicrobial activity to individual HMOs, their effects on biomass production should be investigated.

Importantly, growth inhibition was not maintained across different media, as replacing LB with BHI medium, diminished the 3′-GL inhibitory effect on *S. enterica*. BHI medium is substantially more nutrient-rich than LB and contains a defined carbohydrate source (2–4 g/L dextrose or glucose) (Low et al., 2013; Wijesinghe et al., 2019). Altering the culture medium from nutrient-poor LB to nutrient-rich BHI has been reported to affect planktonic growth, virulence, and glycosylation in *P. aeruginosa* and *S. aureus* (Wijesinghe et al., 2019). The altered planktonic growth may explain the reduced inhibitory effect of 3′-GL on *S. enterica* when switching from LB to BHI medium. Ackerman et al. (2017) also highlighted the importance of nutrient enrichment, showing that 1% glucose supplementation in Todd-Hewitt broth was required for HMOs to inhibit *Acinetobacter baumannii*. On the other hand, the HMO-mediated growth inhibitory response toward *S. agalactiae* strain GB2 disappeared upon the addition of 1% glucose.

Consistent with the influence of nutritional composition, decreasing the NaCl concentration increased the inhibitory activity of GOS against *S. enterica*, while 10 g/L NaCl addition increased the growth of *E. coli* and *Y. enterocolitica*. Although no studies have directly investigated the role for NaCl in modulating oligosaccharide-mediated pathogen growth inhibition, Glover et al. (2022) reported increased glucose metabolism in *E. coli* under salt-depleted conditions. Furthermore, human milk contains 0.2–0.5 g/L NaCl (Bauer and Gerss, 2011), a markedly lower salinity than the 5 g/L used in culture media. As the antimicrobial activity of GOS against *S. enterica* was notably reduced at higher NaCl concentrations (Figure 4E), this may suggest that GOS exert stronger growth-inhibitory effects when *S. enterica* is exposed to oligosaccharides under conditions resembling those in human milk. GOS did not demonstrated detectable antibiofilm activity against the tested bacterial strains (Supplementary Figure 1A). To our knowledge, biofilms of these bacterial species have not been reported to be sensitive to GOS, suggesting that its antibiofilm effects may be species-specific. Although a trend was observed that GOS could increase the sensitivity of *E.coli* toward H_2_O_2_, this was not significant (Supplementary Figure 1B). No change in H_2_O_2_ sensitivity was observed in the other tested bacteria upon GOS treatment, suggesting that GOS do not influence bacterial oxidative stress. Future studies investigating the *in vitro* antimicrobial activity of oligosaccharides may benefit from focusing on functional or metabolic pathways associated with salt stress and osmotic regulation.

Our prepared HMO mixture harbored no growth inhibitory impact against *E. coli, S. enterica* and *Y. enterocolitica*. Considerable growth-inhibitory effects of donor-derived pooled HMO mixtures have been observed against various pathogens (Craft et al., 2018; Lin et al., 2017; Renwick et al., 2025). The HMO mixture used in this study comprised a subset of the most prevalent individual HMOs (9 different structures) at colostrum-like concentration ratios (Soyyilmaz et al., 2021). Given that human milk contains over 200 distinct HMO structures (Bode, 2020), these inhibitory effects may result from specific components or combinations that are absent in our mixture. Evidence from Craft et al. (2018) indicated that donor-derived HMO extracts exhibit distinct growth-inhibitory effects against *S. agalactiae* compared to bottom-up–prepared colostrum-like HMO mixtures. In relation to our observed growth-inhibitory effects of low concentrations of individual HMOs on *S. enterica* and *Y. enterocolitica*, it is possible that different HMOs may modulate or attenuate each other's activity within complex mixtures, or that certain bacteria preferentially interact with specific HMO structures in heterogeneous environments (Renwick et al., 2025; Nakajima et al., 2025). Further investigation of less abundant individual HMOs or fractionated HMO extracts may therefore reveal additional antimicrobial activities that are not apparent in whole HMO mixtures.

In conclusion, HMOs can have distinct growth effects on the growth of bacteria, either promoting or inhibiting, depending on the nutrients composition and salt concentration. 3′-GL and GOS showed the most pronounced growth-inhibitory effects against *Escherichia coli, Salmonella enterica, Yersinia enterocolitica*. Our findings highlight that oligosaccharide structure, bacterial species, and growth environment together shape the observed anti-growth effects of oligosaccharides. Importantly, these observations were obtained under controlled *in vitro* conditions, allowing the direct effects of individual oligosaccharides on bacterial growth to be assessed. As the intestinal environment is considerably more complex, involving host factors, microbial interactions, and dynamic nutrient availability, further studies are needed to determine how these findings translate to *in vivo* settings. Nevertheless, our *in vitro* results suggest a potentially complex but minor antibacterial role for HMOs in protecting the early-life gut from pathogens during disease states such as NEC and gastroenteritis.

## 4 Material and methods

### 4.1 Bacterial strains and growth

Bacterial strains *E. coli* DSM 30083 and *C. perfringens* DSM 756 were purchased from German Collection of Microorganisms and Cell Cultures GmbH (DSMZ, Braunschweig, Germany). *S. enterica* 90-13-706 was obtained from the Centraal Veterinair Instituut (CVI, Wageningen Bioveterinary Research, Lelystad, The Netherlands) and *Y*. enterocolitica W22703, originally isolated and described by Fuchs et al. (2011), was obtained from Wim Gaastra (Infection Biology, Utrecht University, The Netherlands). Growth procedures for the facultative anaerobic pathogens were performed as described by Sigma–Aldrich. (2022). Briefly, *E. coli, S. enterica* and *Y. enterocolitica* were grown in ambient air on Lennox LB (MP Biomedicals, Eschwege, Germany) agar plates, transferred to Lennox LB culture medium and incubated for 16 h at 37°C under 160 rpm shaking conditions. The growth procedure for *C. perfringens* was performed as described by Mavrogeni et al. (2023). Briefly, *C. perfringens* was cultivated on sheep blood agar plates (Analytichem, Mijdrecht, The Netherlands), transferred to BHI culture medium (Becton Dickinson Biosciences, Canada) and incubated for 16 h without shaking at 37° in an anaerobic jar, gassed with an Anoxomat^®^ (Advanced instruments, Norwood, Massachusetts, USA) under anaerobic conditions (0% O_2_/5% H_2_/10% CO_2_/85% N_2_).

### 4.2 Oligosaccharides

The HMOs, 2′-fucosyllactose (2′-FL), 3-fucosyllactose (3-FL), 3′-sialyllactose (3′-SL), 6′-sialylactose (6′-SL), lacto-N-tetraose (LNT), lacto-N-neotetraose (LNnT), 3′-galactosyllactose (3′-GL), 4′-galactosyllactose (4′-GL), 6′-galactosyllactose (6′-GL), and galacto-oligosaccharides (GOS) were provided by Friesland Campina (Amersfoort, The Netherlands) (see Table 1 for source and purity information).

**Table 1:** Human milk oligosaccharides used in this study with supplier and purity information.

Component	Supplier	Purity
2′-FL	FrieslandCampina	98%
3-FL	Elicityl	>95%
3′-SL	Biosynth	>98%
6′-SL	Biosynth	>92%
LNT	FrieslandCampina	>90%
LNnT	Elicityl	>95%
3′-GL	Synvenio	>90%
4′-GL	Synvenio	>95%
6′-GL	Synvenio	>98%
GOS	FrieslandCampina	>90%

### 4.3 Bacterial growth curves

Prior to bacterial growth measurements, filter sterilized HMO-stock solutions (20 mg/ml) dissolved in culture medium were serially diluted in a 96-well plate ending with a 150 μL volume per well. *E. coli, S. enterica* and *Y. enterocolitica* were aerobically grown in Lennox LB medium (Analytichem, Mijdrecht, The Netherlands) or Bacto^TM^ Brain heart infusion (BHI) broth (Becton Dickinson Biosciences, Canada) as previously described. A 16-hour incubated liquid culture was diluted to an initial OD600nm of 0.01 in 150 μL of Lennox LB or BHI medium (both 5 g/L NaCl), either supplemented with or without 0.25, 0.5, 1 or 2% oligosaccharides and/or varying NaCl concentration of 0, 0.5, 2.5, 5, 10 g/L.

Growth was measured as described by Buzun et al. (2024). using a BioTek Synergy HTX Multimode Reader (Agilent, Santa Clara, California, USA) with OD600nm readings every 10 min for 16 h at 37°C in ambient air under continuous orbital shaking at 283 cpm. *C. perfringens* was anaerobically grown in BHI as previously described. A 96 well plate with BHI either supplemented with or without 0.25, 0.5, 1 or 2% oligosaccharides was anaerobically incubated for at least 1 h prior inoculation. Growth was measured using an Alto Microplate reader (Cerillo, Charlotteville, Virginia, USA) with optical density (OD600nm) readings every 10 min for 16 h at 37°C under anaerobic conditions in an anaerobic jar without shaking. All OD600nm measurements were performed in triplets, subtracted by the uninoculated blanks per growth condition.

### 4.4 Colony forming unit determination

The colony forming units (CFUs) was performed using the track dilution method and were determined after 0h, 4h, 8h, 24h, and 48h growth (Wise, 2006). Growth conditions were identical to those used for the growth curves, except scaled to a volume of 500 μL in a 48-well plate. The 48-well plate was incubated at 37°C under ambient air, shaken at 160 rpm between sample collections. At each interval, 10 μL was serial diluted in PBS and plated on Lennox LB agar. After overnight incubation at 37°C the colonies were counted.

### 4.5 Growth curve analysis

The growth curves were analyzed to statistically compare the parameters lag-time (h), onset of the stationary phase (h), maximum growth rate (OD600nm/h) and maximum optical density measurement (OD600nm) between HMO-treated and untreated cultures. The parameters were adapted from the algorithm described by Navarro-Pérez et al. (2022) OD600nm measurements were taken every 10 min. Lag-time was defined as the first time point at which three consecutive second derivatives statistically differed from zero. To calculate the maximum growth rate (μmax/h), OD600nm values in the exponential region were natural log–transformed and plotted against time starting from the previously determined lag-time. A linear regression was fitted to ln (OD600nm) vs. time, and the slope of this line was taken as μmax. The stationary phase was identified as the first time point of three consecutive first derivatives, after inflection (specific time point at maximum specific growth rate). The maximum optical density measurement represented the highest OD600nm value observed during 16 h of growth.

Experiments were performed in triplicates and repeated in at least three independent experiments. Data was processed with Excel.

### 4.6 Bacterial biofilm assay

Bacterial biofilm formation was assessed using an adapted version of the procedure described by Craft et al. (2018). Briefly, *E. coli, S. enterica*, and *Y. enterocolitica* were cultured overnight at 37°C and 160 rpm in Lennox LB medium as described in Aliquots of 300 μL were transferred in duplicate to glass tubes, including non-inoculated blank controls, and incubated for 24 h at 37°C under static conditions in ambient air. Following incubation, the planktonic cell suspension was removed, and the tubes were gently washed once with phosphate-buffered saline (PBS) to remove non-adherent cells. The remaining adherent cells were stained with 600 μL of 0.5% crystal violet solution for 30 min. Subsequently, the tubes were washed six times with PBS to remove excess stain and allowed to dry overnight at room temperature. To quantify biofilm biomass, the bound crystal violet was solubilized with 500 μL of an 80% ethanol/20% acetone solution, and absorbance was measured at OD595nm using a spectrophotometer. Absorbance values were corrected by subtracting the corresponding blank measurements.

### 4.7 Hydrogen peroxide-mediated killing

To assess oxidative stress, the hydrogen peroxide-mediated killing assay described by Nong et al. (2026) was adapted and applied to the facultative anaerobic bacterial pathogens included in this study. Briefly, *E. coli, S. enterica*, and *Y. enterocolitica* were cultured overnight at 37°C and 160 rpm in Lennox LB medium as described in Section 4.1. Overnight cultures were subsequently diluted 1:1000 in fresh Lennox LB medium and grown to the exponential phase. Cells were then harvested by centrifugation, washed, and resuspended in phosphate-buffered saline (PBS) to a final OD600nm of 0.2. Aliquots of 250 μL of bacterial suspension were exposed in duplicate to 0, 5, 10, or 50 mM H_2_O_2_. After incubation for 10 or 30 min, 10 μL samples were serially diluted and plated in duplicate on Lennox LB agar plates. Plates were incubated overnight at 37°C, after which colony-forming units (CFUs) were enumerated and expressed as CFU/mL.

### 4.8 Statistics

Statistical analysis was conducted with an one-way analysis of variance (ANOVA) followed by a Dunnett's multiple comparisons test or two-way ANOVA followed by a Holm-Šídák's multiple comparisons test in GraphPad^®^ version 10.4. Statistical significance is indicated by *p*-value markers: ^*^, *p* < 0.05; ^**^, *p* < 0.01; ^***^, *p* < 0.001; ^****^, *p* < 0.0001.

## Data Availability

The raw data supporting the conclusions of this article will be made available by the authors, without undue reservation.
